# Triage Strategies for Non-16/Non-18 HPV-Positive Women in Primary HPV-Based Cervical Cancer Screening: p16/Ki67 Dual Stain vs. Cytology

**DOI:** 10.3390/cancers15205095

**Published:** 2023-10-21

**Authors:** Karolina Mazurec, Martyna Trzeszcz, Maciej Mazurec, Joanna Streb, Agnieszka Halon, Robert Jach

**Affiliations:** 1 Corfamed Woman’s Health Center, Kluczborska 37, 50-322 Wroclaw, Poland; m.trzeszcz@corfamed.pl; 2 Division of Pathology and Clinical Cytology, University Hospital in Wroclaw, Borowska 213, 50-556 Wroclaw, Poland; 3 Department of Oncology, Jagiellonian University Medical College, Kopernika 50, 31-501 Krakow, Poland; joanna.streb@uj.edu.pl; 4 Department of Clinical and Experimental Pathology, Division of Clinical Pathology, Wroclaw Medical University, Borowska 213, 50-556 Wroclaw, Poland; agnieszka.halon@umed.wroc.pl; 5 Division of Gynecologic Endocrinology, Jagiellonian University Medical College, Kopernika 23, 31-501 Krakow, Poland; jach@cm-uj.krakow.pl

**Keywords:** cervical cancer screening, high-risk HPV, p16/Ki67 dual staining, DS, prevention, triage

## Abstract

**Simple Summary:**

Cervical cancer is often caused by certain high-risk types of human papillomavirus (HPV), mainly types 16 and 18. However, there are 12 other high-risk HPV types that can also increase the risk of cancer. When these non-16/non-18 high-risk HPV types are detected (HPV HR12+), additional tests are needed to assess the risk of precancerous changes and cancer and avoid unnecessary treatments. Currently, two triage tests, cytology and p16/Ki67 dual-stain (DS), are being considered. In our study, we looked back at how well three different screening strategies worked: M1—using cytology, M1A—using cytology with DS in selected cases, and M2—using DS in all cases. We found that using DS as a triage test was more effective than cytology alone in selected HPV HR12+ cases. It allowed us to find more cases of high-grade precancerous changes (HSIL+) while reducing the need for unnecessary colposcopies.

**Abstract:**

Background: In the context of primary HPV cervical cancer screening, the identification of minor screening abnormalities necessitates triage tests to optimize management and mitigate overtreatment. Currently, reflex cytology and reflex p16/Ki67 dual-stain (DS) are under scrutiny for their applicability in primary HPV-based screening. However, there remains a dearth of comprehensive data for comparing their performance. Methods: Among 30,066 results from liquid-based cervical cancer screening tests, a cohort of 332 cases was meticulously selected based on available high-risk human papillomavirus (HPV) test results, limited genotyping for HPV 16 and 18, liquid-based cytology, DS, and histology outcomes from standardized colposcopy with biopsy. For cases positive for 12 other high-risk HPV genotypes, three retrospective triage approaches were analyzed. We computed the positive predictive value (PPV) for the detection of high-grade squamous intraepithelial lesions or worse (HSIL+). Results: Both triage models employing DS (reflex cytology followed by DS and reflex DS alone in all cases) exhibited significantly higher PPV for HSIL+ compared to the strategy with reflex cytology alone (35.9%/33.3% vs. 18.8%; *p* < 0.0001). Additionally, these DS-based models showed higher negative predictive values (NPV) (100%/96.2% vs. 69.2%; *p* = 0.0024/0.0079). In the DS-inclusive models, fewer colposcopies were necessitated (103/102 vs. 154), and fewer cases of HSIL+ were overlooked (0/3 vs. 8). Conclusions: Our findings suggest that p16/Ki67 dual-stain, either as a standalone or combined triage test, holds promise for the effective detection of HSIL+ in patients with minor screening abnormalities in primary HPV-based cervical cancer screening.

## 1. Introduction

Cervical cancer, estimated at 604,127 new cases and 341,831 deaths in 2020, ranks as the fourth most common cancer among women worldwide [1]. In developed nations, the incidence and mortality of cervical cancer have significantly declined over recent decades, primarily attributed to widespread and effective screening and vaccination initiatives [2].

The development of cervical cancer is closely linked to persistent infection with 13–14 high-risk types of the human papillomavirus (HRHPV) [3,4,5]. Currently, molecular detection of these specific HPV types is the globally recommended primary screening method [6,7,8]. Primary HPV-based cervical cancer screening programs aim to identify precancerous lesions at an early stage, preventing their progression to cervical cancer with remarkable sensitivity [9,10]. However, the trade-off is a potential issue of overdiagnosis and overtreatment due to reduced specificity [11]. Consequently, minimizing overtreatment is pivotal to enhancing screening effectiveness while ensuring patient safety and comfort.

The accuracy of screening tests varies based on various factors, with HRHPV-positivity being a critical determinant [12]. The type of HPV detected influences the risk of histologic high-grade squamous intraepithelial lesions, including quantification of cervical intraepithelial neoplasia grade 2 or worse (HSIL/CIN2+). Among these HPV types, HPV 16 carries the highest risk [13]. Thus, HRHPV genotyping, even when limited to HPV 16 and 18, can significantly contribute to risk stratification.

According to the 2019 American Society for Colposcopy and Cervical Pathology risk-based guidelines (ASCCP 2019), as well as many other international guidelines, positive results for HPV 16 and/or 18 (HPV 16/18+) are considered significant screening abnormalities. Regardless of cytological results, individuals with HPV 16/18+ are recommended to undergo colposcopy to rule out the presence of high-grade precancerous lesions or cancer [4,14]. However, for individuals testing positive for 12 other high-risk HPV types, excluding HPV 16 and 18 (HPV HR12+), the need for triage tests is evident to establish the most appropriate management strategy [15,16,17]. Initially, cytology served as the primary triage test in HPV-based screening strategies due to its widespread availability [18,19]. In 2020, a new diagnostic tool received approval from the US Food and Drug Administration. This tool is used as a triage test in primary HPV-based screening for HPV-positive cases and for managing abnormal screening results in primary cotesting. This new tool, known as p16/Ki67 dual-staining, is an immunocytochemical test that utilizes cervical cytology specimens. It concurrently assesses anti-proliferative p16 and Ki67 proliferative proteins [20,21]. Notably, it is characterized by both high sensitivity and specificity in detecting HSIL/CIN2+ [22,23,24,25].

Minor screening abnormalities often signify the presence of HRHPV infection, with a potentially lower risk of HSIL/CIN2+. Consequently, these cases require special attention in primary HPV-based cervical cancer screening to prevent overdiagnosis and overtreatment. In such instances, immediate colposcopy may not be necessary, and primary HRHPV testing with a follow-up in one year is the recommended approach. According to the ASCCP 2019 guidelines, if the immediate risk of histologic high-grade squamous intraepithelial lesions with a quantification of cervical intraepithelial neoplasia in grade 3 or worse (HSIL/CIN3+) is below 4% and the cumulative 5-year risk of HSIL/CIN3+ is equal to or above 0.55%, a repeat HRHPV test in one year is advisable [4].

Predictive values hold significant importance for clinicians, surpassing sensitivity and specificity in their value, as they provide an estimate of the likelihood that a test correctly identifies the presence or absence of a disease based on its results [26]. Among these, the positive predictive value (PPV) stands as a critical parameter in evaluating the accuracy of diagnostic tests for detecting HSIL/CIN2+ in women with minor screening abnormalities—potentially indicating a lower risk of HSIL/CIN2+. The PPV signifies the likelihood that a positive test result accurately predicts the presence of the disease. If a highly sensitive test predicts a patient as negative for a particular condition, it is reasonable to exclude that condition for the patient [27]. The PPV, however, varies depending on factors such as the prevalence of the condition in the screened population, the sensitivity (typically very high for primary HPV), and the specificity (usually lower for primary HPV) of the test [28]. Women with minor screening abnormalities indeed face a higher risk of developing HSIL/CIN2+ compared to those with negative results [29]. Still, the extent of this risk varies based on the test results and other contributing factors. Unfortunately, the PPV of minor screening abnormalities for the development of HSIL/CIN2+ remains inadequately established. Limited data are available concerning the performance of the p16/Ki67 biomarker in triaging these cases, especially when used in conjunction with limited 16/18 genotyping.

Hence, the objective of our retrospective study is to assess the predictive values of various p16/Ki67 dual staining and cytology triage models, either individually or in combination, for detecting HSIL/CIN2+ in women exhibiting minor screening abnormalities. These women underwent primary HPV-based cervical cancer screening with limited HPV 16/18 genotyping and received HPV HR12+ positive results.

## 2. Materials and Methods

### 2.1. Study Population

**Figure 1:**
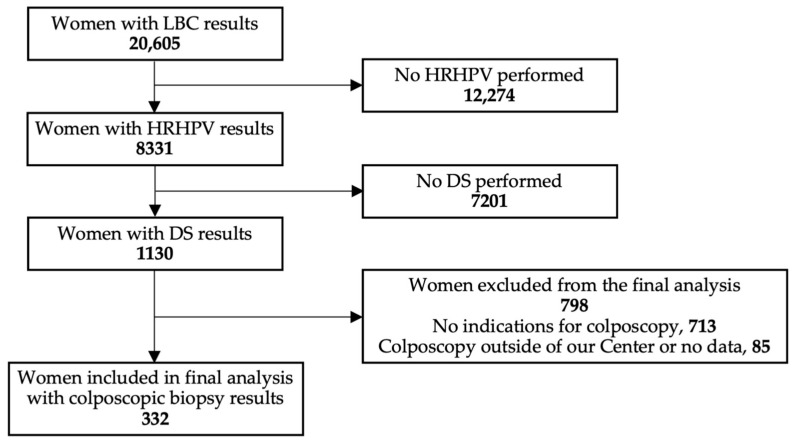
Flowchart reporting study group selection. Abbreviations: LBC, liquid-based cytology; HRHPV, 14 high-risk types of human papillomavirus test; DS, p16/Ki67 dual staining test.

Our study is a post-hoc analysis and included a total of 30,066 cervical cancer liquid-based screening (LBS) test results, comprising 20,605 cases of liquid-based cytology (LBC), 8331 cases of HRHPV test results with limited HPV 16/18 genotyping, and 1130 cases of DS. The analysis was conducted on LBS test results obtained from August 2015 to July 2020 in an opportunistic private funds-based cervical cancer screening at one of the largest private outpatient gynecological clinics in Lower Silesia in Poland (Corfamed Woman’s Health Center). The screening tests were collected from women aged 15 to 92. During the analyzed period, the Center used three cervical cancer screening strategies: primary cytology with reflex HRHPV, primary cotesting, and primary cotesting plus (cotesting simultaneously performed with DS). Screening algorithms used in the center during the study were described previously [30]. The final study group consisted of 332 patients over 25 years old who had all three LBS test results and a colposcopy with biopsy performed in our center (Figure 1). The selected group of patients had an unknown previous HPV status. A retrospective analysis of cytologic-virologic-immunocytochemical test results was carried out along with histology, and the predictive values of cytology and DS as a triage test in selected minor cervical cancer screening abnormalities were evaluated.

Minimally abnormal cervical cancer screening test results are a category for HRHPV-positive tests with reflex cytology negative for intraepithelial lesion or malignancy (NILM) or minor cytologic abnormalities—atypical squamous cells of undetermined significance (ASC-US) or low-grade squamous intraepithelial lesion (LSIL) [31]. Selected minor screening abnormalities/minimally abnormal test results used in the study are presented in Table 1 [4,14,31,32].

The methodology was described thoroughly previously, as the study is part of a series [30,34]. The ethics committee approved (ID: 118.6120.36.2023).

### 2.2. HRHPV Testing

The Abbott RealTime High-Risk HPV molecular in vitro PCR test (Abbott Molecular, Des Plaines, IL, USA) was used to detect HRHPV. According to a laboratory report, it was performed following the manufacturer’s recommendations. The test phenotypes 12 types of high-risk HPV DNA (31, 33, 35, 39, 45, 51, 52, 56, 58, 59, 66, and 68) and genotypes HPV 16 and 18 (limited genotyping). The test results were categorized as HPV 16/18+ if either of these types was detected, while the result was classified as HPV HR12+ if one or more of the other twelve non-16 and non-18 HRHPV types were detected. All screening samples were collected using the Cervex-Brush device (Rovers Medical Devices, Oss, The Netherlands), and all screening tests (HRHPV, LBC, and DS) were carried out in the same laboratory.

### 2.3. Liquid-Based Cytology

An external laboratory prepared liquid-based SurePath slides using the automatic PrepStain Slide system (Becton Dickinson, Franklin Lakes, NJ, USA) in strict accordance with the manufacturer’s protocol. The laboratory stored all residual cervical samples for three months under specified conditions, enabling additional tests to be performed when indications occurred (including DS triage) without the need for additional gynecological appointments. This approach allowed cervical cancer diagnostics to be performed from a single cervical material sample collection. A gynecological cytopathologist assessed all cytology samples and classified them according to the Bethesda 2014 system, being aware of the HRHPV status of the cervical samples. The evaluation of quality and control procedures was based on benchmarks released by US laboratories that were accredited by the College of American Pathologists; the study’s reporting rates remained within the normal ranges reported [30].

### 2.4. p16/Ki67 Dual-Stain Testing

A CINtec PLUS detection kit (Roche, MTM AG laboratories, Munich, Germany) was used for dual immunocytochemical staining with p16 and Ki67 proteins and was processed in an automated BenchMark XT system (Ventana Medical Systems, Inc., Oro Valley, AZ, USA) following the manufacturer’s protocol, as provided by the laboratory coordinator. In each run, a control specimen was present. The DS was carried out using residual cellular material from the original SurePath vials (Becton Dickinson, Franklin Lakes, NY, USA), which had been stored in the laboratory after cytology and/or HPV testing. A qualified gynecological pathologist, who had also evaluated the LBC, assessed the DS, and reported it as positive/negative/unsatisfactory, followed the available definitions [35].

### 2.5. Colposcopy Protocol

The cases that were DS-positive, ASC-US or LSIL HRHPV-positive, or with cytologic ASC-H or worse, regardless of/without HRHPV status, were referred for a colposcopy with biopsy, according to the Polish recommendations with the extension of the 2012 ASCCP guidelines and the 2015 ASCCP interim guidelines [17,36,37,38]. According to the Polish protocols, the minimal colposcopy protocol requirements included endocervical sampling (curettage and brushing), direct biopsies for all colposcopy abnormal findings or suspicious for invasion, or additional random biopsies. A gynecological pathologist reviewed all histologic diagnoses of cervical biopsies and endocervical sampling based on the LAST 2012/WHO 2014 terminology [39,40]. The study did not include results from colposcopies performed outside the Center or histologic reports evaluated by pathologists other than the Center’s gynecological pathologist, as they may have used different colposcopy protocols, different histologic nomenclature, and/or no recommended p16 immunohistochemistry in their reports.

### 2.6. Triage Models Analyzed

**Figure 2:**
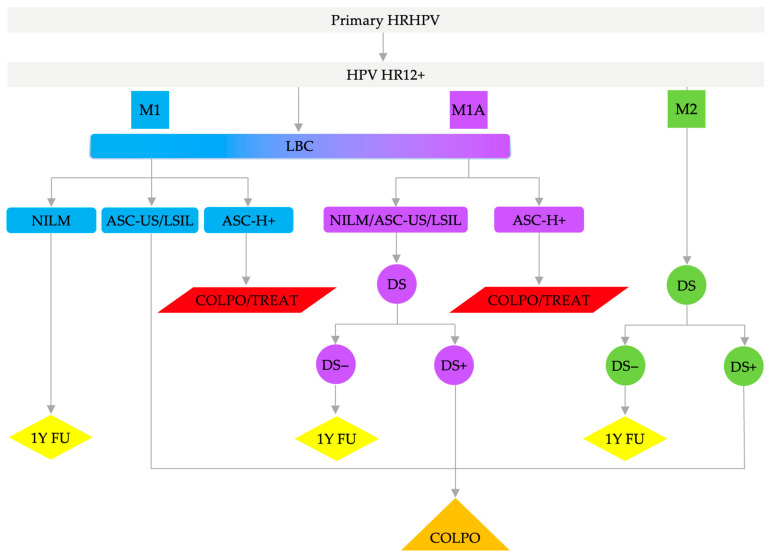
Analyzed triage models—flowchart. Abbreviations: HRHPV, 14 high-risk types of human papillomavirus test; HPV HR12+, human papillomavirus 12 high-risk types other than types 16 and 18 positive results; M1, Triage model 1; M1A, Triage model 1A; M2, Triage model 2; LBC, liquid-based cytology; NILM, negative for intraepithelial lesion or malignancy; ASC-US, atypical squamous cells of undetermined significance; LSIL, low-grade squamous intraepithelial lesion; ASC-H, atypical squamous cells—cannot exclude HSIL; ASC-H+, ASC-H or worse; DS, p16/Ki67 dual staining test; +, positive; −, negative; 1Y FU, 1-year follow-up; COLPO, colposcopy; COLPO/TREAT, colposcopy/treatment.

In the study, we have analyzed and compared three HSIL/CIN2+ risk triage models for HPV HR12+ cases in primary HPV-based cervical cancer screening. In model 1 (M1), a reflex cytology was used in all positive cases; in model 1A (M1A), a reflex cytology was carried out in all positive cases combined with DS performed after NILM, ASC-US, or LSIL diagnoses as the second triage test; and in model 2 (M2), a reflex DS was used in all HPV HR12+ cases. The management steps in the studied triage models are shown in Table 2 and in Figure 2.

### 2.7. Statistical Analysis

The diagnostic value of the screening tests used and their combinations was determined by the positive predictive value (PPV) and negative predictive value (NPV), calculated based on the standard definition.

In our study, we evaluated the PPV of HSIL/CIN2+ risk triage tests in women with minor screening abnormalities in primary HPV-based cervical cancer screening with limited genotyping. The PPV was calculated by dividing the number of women with positive test or test combination results and confirmed HSIL/CIN2+ in colposcopic biopsy by the total number of women with positive test or test combination results.

The discrepancies in the diagnostic value between the screening test variations were assessed using the Leisenring et al. method [41] with the use of the DTComPair package in R. Statistical significance was set at *p* < 0.05. The statistical analysis was performed using licensed PQStat software in the 1.6.0 full version (2015 PQStat Statistical Calculation Software, Poznan, Poland).

## 3. Results

Among 30,066 cervical cancer liquid-based screening test results, including 20,605 LBC, 8331 HRHPV, and 1130 DS, 443 cases had colposcopy with biopsy performed at our center. Out of those, 332 women over 25 years old were selected as eligible for the study, as shown in Table 3. The age range was 25 to 67 years old, with a median age of 33 and an average age of 34.7 years. Women <25 years old were not included in the final study group.

All selected cases were HRHPV-positive, of which 45.8% (n = 152) were HPV 16/18+ and 54.2% (n = 180) were HPV HR12+. Of all HPV HR12+ cases, 56.7% (n = 102) were DS-positive, and 43.3% (n = 78) were DS-negative. In the HPV HR12+ group, 166 cases had negative cytology (NILM) or minor cytologic abnormalities (ASC-US or LSIL) [14.4% (26/180) NILM, 34.4% (62/180) ASC-US, and 43.3% (78/180) LSIL cases], and 14 cases had major cytologic abnormalities (ASC-H or worse) [7.8% (14/180)]. The histology results in the HPV HR12+ group were negative, or LSIL/CIN1 was diagnosed in 78.9% (n = 142) of cases, and HSIL/CIN2+ was detected in 21.1% (n = 38), of which 31.6% (12/38) of cases were HSIL/CIN3+. In further analysis, we have focused on HSIL/CIN2+. The group characteristics are presented in Table 4.

In the analysis of the study group in M1, 29 HSIL/CIN2+ cases were detected, which required 154 colposcopies; 8 cases were missed. In M1A, respectively, 37/103/0, and in M2, 34/102/3. The missed HSIL/CIN2+ cases resulted from the lack of indications for colposcopy based on the algorithms applied to each of them. Complete results with PPV values for the results combinations and for the three analyzed models are presented in Table 5.

There was a statistically significant difference in PPV between triage models M1 and both M1A and M2: 18.8% vs. 35.9% (*p* < 0.0001) and 18.4% vs. 33.3% (*p* < 0.0001), respectively. The difference in PPV between models with the incorporation of DS, M1A, and M2 was not statistically significant (*p* = 0.0811). There was also a statistically significant difference in NPV between triage models M1 and both M1A and M2, 69.2% vs. 100.0% (*p* = 0.0024) and 69.2% vs. 96.2% (*p* = 0.0079), respectively. The difference in NPV between M1A and M2 triage models was not statistically significant (*p* = 0.0773). The details of this comparison are shown in Table 6 and Table 7.

The triage models in primary HPV-based screening were also compared by the number of colposcopies required per HSIL/CIN2+ detection and the number of missed HSIL/CIN2+ cases. The number of colposcopies needed to detect HSIL/CIN2+ was on average nearly two times lower in the triage model with DS as the second triage after cytology (M1A) and DS alone (M2) than in the model involving cytology only (M1). In M1, 8 HSIL/CIN2+ cases out of 154 performed colposcopies were missed, while only 3 HSIL/CIN2+ cases out of 102 colposcopies were missed in triage with DS only (M2) and none out of 103 in triage model with DS after cytology (M1A). In the screening model 0 (M0), which was a hypothetical comparative model in which all HPV HR12+ cases were referred directly to colposcopy, 180 colposcopies were required (14% fewer colposcopies in M1 and approximately 43% fewer in both M1A and M2), and approximately 1.6–1.7 times more colposcopies were needed to detect 1 HSIL/CIN2+ case than in M1A and M2 (Table 8).

## 4. Discussion

This is the first study to evaluate the diagnostic performance of different triage models in patients positive for 12 non-16/non-18 HRHPV types who have been screened with primary HPV with limited HPV 16/18 genotyping, including incorporation of p16/Ki67 as a standalone triaging test or as the second triage test after cytology in selected cases. The analysis may help to compare the most widely used worldwide cytologic triage, the well-known diagnostics, with a newly proposed option of triage with a biomarker of transforming infection in women with a lower risk of HSIL/CIN2+ who need effective management and a proper selection for colposcopy. In the study, both triage models with reflex DS (M1A and M2) had significantly higher PPVs for detection of HSIL/CIN2+ than triage model with reflex cytology alone (M1) (35.9% vs. 18.8%, *p* < 0.0001 and 33.3% vs. 18.8%, *p* < 0.0001). That means patients with positive test results in M1A and M2 are more likely to be HSIL/CIN2+ when test combination results are positive, which justifies more accurate colposcopy referrals in these cases. In addition, triage models with incorporation of DS after cytology or alone (M1A and M2) result in a about 30% reduction in the number of colposcopies in comparison to cytologic triage M1 (103/102 vs. 154). M1A had a higher PPV than M2, but the difference was not statistically significant (*p* = 0.0811), suggesting that there is no advantage of triaging with DS after cytology over DS alone triage in terms of PPV—it prevents unnecessary colposcopies to the same extent, as demonstrated in the number of colposcopies needed (103 in triage with DS after cytology vs. 102 in triage with DS alone). The highest PPV achieved for analyzed triage models in women with minor screening abnormalities for detection of HSIL/CIN2+ in our study was 41.4% for HPV HR12+ ASC-US DS-positive cases in triage with DS after cytology (M1A), followed by 33.7% for HPV HR12+ NILM/ASC-US/LSIL DS-positive test result also in M1A and 33.3% for HPV HR12+ with DS-positive test in triage with DS alone (M2). The NPV of models with DS triage was very high (96.2–100.0%), assuring high safety for patients in both triaging approaches. Differences between these and cytologic triage M1 (NPV 69.2%) were statistically significant (*p* = 0.0024 and *p* = 0.0079, for M1A and M2, respectively). While the number of missed HSIL/CIN2+ cases was higher in triage with DS alone than in triage with DS after cytology (three missed cases in M2 vs. zero missed cases in M1A), it was almost three times lower than in the currently predominant worldwide triage with cytology alone (eight missed cases in M1). In M1, 78.4% of HSIL/CIN2+ cases were detected, 91.9% in M2, and 100% in M1A. Due to the relationship between PPV and disease prevalence, it may differ across populations [42]. However, all the PPV obtained met the criterion of a value greater than 10% for an acceptable triage strategy, according to data recently reported by Hammers et al. [43].

In comparison to the hypothetical model without triage (M0), the number of colposcopies needed was significantly lower in all analyzed triage models (14% fewer colposcopies needed in M1 and around 43% fewer colposcopies in both triage models with the incorporation of DS—M1A and M2). Nevertheless, it did not reflect the actual number of required colposcopies in the strategy without a triage test. In our study, only a small number of NILM results were included (with indications for colposcopy), as colposcopy referral in all HPV HR12+ cases is unacceptable due to the very high risk of overtreatment [16,17]. According to data from our center, the NILM results were 54.8% of HPV HR12+ cases, which indicates the approximate number of colposcopies needed in M0 [33].

The results achieved for selected sets of results were similar to many other studies, including data reported by Øvestad et al. with nearly identical PPV for detection of HSIL/CIN2+ in HPV HR12+ women with DS triage (33.3% vs. 37.2%, respectively) and NPV for the same group (96.2% vs. 91.8%, respectively) [44]. In turn, our results demonstrated higher PPV for detection of HSIL/CIN2+ in HPV HR12+ cases with reflex DS compared with the US studies (33.3% vs. 19.2%, respectively), but nearly equal NPV was found (96.2% vs. 96.4%, respectively) [21]. Similar differences in predictive values for results in HPV HR12+ women with DS triage were obtained by Wright et al. (PPV 19.6% and NPV 96.4%), while PPV for HSIL/CIN2+ in HPV HR12+ cases with cytology triage was very similar to our analysis (17.8% vs. 18.8%) [45]. As this is one of the first population-based studies comparing a 2-level triage strategy performed with DS after cytologic triage in cases with NILM, ASC-US, or LSIL results, a reliable study comparator for predictive values obtained for detection of HSIL/CIN2+ in HPV HR12+ women in the M1A triage model was not found. The PPV for HSIL/CIN2+ obtained by Ebisch et al. for HRHPV+ cases triaged with DS+, ASC-US+, or NILM/ASC-US/LSIL DS+ were much higher than for similar cases in our study (HPV HR12+ cases with the same triage) (74% vs. 33.3%, 69% vs. 18.8%, and 74% vs. 33.7%, respectively), the same as by El-Zein et al. for HRHPV+ cases (29.7% vs. 20.6% in our analysis for HPV HR12+ cases) [46,47]. Comparing our results with studies evaluating all types of HRHPV together, the PPV in our analysis was relatively small, which was caused by excluding HPV 16/18+ cases representing major screening abnormalities and thus did not meet the criteria of our study.

The highest PPV in our study was in M1A (DS triage after cytologic triage), but the difference between this result and PPV in the triage approach with DS alone (M2) was not statistically significant. Since M2 requires only one triage test after primary HRHPV, that is reflex DS alone, and M1A needs not only reflex DS but also reflex cytology in the first triaging level, M2 should be the most effective model in detecting HSIL/CIN2+. These findings support the use of DS as a triage strategy for women with selected minor screening abnormalities in the primary HPV-based cervical cancer screening strategy, as it can improve the accuracy of the screening and reduce the need for colposcopy. However, further studies are needed to confirm these findings.

Our results are consistent with previous studies that have shown that DS improves the accuracy of cervical cancer screening for women with minor screening abnormalities [22,44]. The study also showed that DS had a higher PPV than cytology for the detection of HSIL/CIN2+. However, the PPV of DS can vary depending on the population being tested, the prevalence of the disease, and the threshold used for defining a positive test result. Therefore, it is important to evaluate the PPV in the context of the population being tested. The results of the age group <25 years old were close to the results of the 25+ group, but due to the small size of this group, they were not statistically significant, and for this reason, they were not included in the study.

This study has several strengths. One of the most important is a joint analysis of all cervical cancer screening tests locally approved, including liquid-based cytology, HRHPV testing, and DS, which allowed us to compare the accuracy of these tests and evaluate their potential for use as a triage strategy for HPV HR12-positive women with minor screening abnormalities. All LBCs and DSs were evaluated by a qualified gynecologic cytopathologist. Other strengths were the analysis of the largest number of liquid-based screening results in Poland and Central Eastern Europe countries, a wide age range of participants, and insight into private-based opportunistic cervical cancer screening test results. The study is also one of the largest studies on cytologic-virologic-immunocytochemical-histologic correlations in cervical cancer screening. On the other hand, the study has limitations, such as being a post-hoc analysis. Not all patients with abnormal screening results underwent colposcopy with a biopsy at the center. Due to the different histologic terminology and/or colposcopic protocols and/or the lack of p16 stain in cervical histologic specimens, the study did not incorporate the results of colposcopic biopsies carried out outside the facility. One of the other major limitations is the sample size, which may have affected the accuracy of the PPV estimate. Our study was conducted in a single, private-funds-based center, and the results may not be generalizable to other populations. Future studies with larger sample sizes and multicenter designs are needed to confirm our findings.

## 5. Conclusions

In conclusion, the utilization of the DS triage test emerges as a highly effective approach for the detection of HSIL/CIN2+ in selected cases characterized by minor screening abnormalities in primary HPV-based cervical cancer screening. Our study underscores that in women with HPV HR12-positive results and concurrent DS positivity, referral to colposcopy is a suitable course of action due to the substantial risk of HSIL/CIN2+ in these instances. Conversely, the notably low prevalence of DS-negative cases with HSIL/CIN2+ suggests that they may be safely managed with follow-up screening rather than immediate colposcopy, thereby reducing the number of unnecessary procedures. Consequently, the DS stands as a valuable tool, poised to enhance the precision and efficiency of primary HPV-based cervical cancer screening programs.

## Figures and Tables

**Table 1:** Triage test recommendations for selected minor screening abnormalities/minimally abnormal test results (HPV HR12+) (with unknown prior screening results or HRHPV-negative test within the last 5 years) and recommendations for 1-year follow-up according to ASCCP 2019 Risk-Based Management Consensus Guidelines [4,14,31,32].

**Current HRHPV Result**	**Triage Test**	Triage Test Result	Screening History	**Management Recommendation**
HPV HR12+	Reflex cytology	NILM	Unknown	1-year follow-up with primary-HRHPV
	Reflex cytology	ASC-US or LSIL	HRHPV-negative within last 5 years	
	Reflex DS *	DS-negative	Noncontributory	

Abbreviations: HRHPV, 14 high-risk types of human papillomavirus test; HPV HR12, human papillomavirus 12 high-risk types other than types 16 and 18; DS, p16/Ki67 dual staining test; NILM, negative for intraepithelial lesion or malignancy; ASC-US, atypical squamous cells of undetermined significance; LSIL, low-grade squamous intraepithelial lesion; +, positive. * In preparation for implementation in the US at the end of 2023 [33].

**Table 2:** The analyzed models—summary of the evaluated management.

M1: HPV HR12+ with reflex cytology	NILM—1 y follow-up	ASC-US/LSIL—colposcopy	ASC-H+—colposcopy/treatment
M1A: HPV HR12+ with reflex cytology, combined with reflex DS in NILM/ASC-US/LSIL	DS-negative—1 y follow-up	DS-positive—colposcopy	ASC-H+—colposcopy/treatment
M2: HPV HR12+ with reflex DS	DS-negative—1 y follow-up	DS-positive—colposcopy	

Abbreviations: M1, Triage model 1; M1A, Triage model 1A; M2, Triage model 2; HPV HR12+, human papillomavirus 12 high-risk types other than types 16 and 18 positive results; DS, p16/Ki67 dual staining test; NILM, negative for intraepithelial lesion or malignancy; ASC-US, atypical squamous cells of undetermined significance; LSIL, low-grade squamous intraepithelial lesion; ASC-H, atypical squamous cells—cannot exclude HSIL; ASC-H+, ASC-H or worse.

**Table 3:** The study group selection.

	<25 y, No.	≥25 y, No.	Total, No.
LBC + HRHPV + DS + CB	30	413	443
LBC + HRHPV+ + DS + CB	22	332 *	355
LBC + HPV HR12+ + DS + CB	15	180	195
NILM/ASC-US/LSIL + HPV HR12+ + DS + CB	14	166	180

Abbreviations: LBC, liquid-based cytology; HRHPV, 14 high-risk types of human papillomavirus test; DS, p16/Ki67 dual staining test; CB, colposcopy with biopsy; HPV HR12, human papillomavirus 12 high-risk types other than types 16 and 18; NILM, negative for intraepithelial lesion or malignancy; ASC-US, atypical squamous cells of undetermined significance; LSIL, low-grade squamous intraepithelial lesion; +, positive; y, years; * final study group.

**Table 4:** Study group characteristics.

	Total, No.
LBS (HRHPV, LBC and DS) + histology results	332
HPV 16/18+—major screening abnormalities	152 (45.8%)
HPV HR12+—screening abnormalities with unknown significancy	180 (54.2%)
Results of cytology triage in HPV HR12+ cases	180
Minor screening abnormalities	
NILM	26 (14.4%)
ASC-US	62 (34.4%)
LSIL	78 (43.3%)
Major screening abnormalities	
ASC-H+	14 (7.8%)
Results of DS triage in HPV HR12+ cases	180
DS-positive—major screening abnormalities	102 (56.7%)
DS-negative—minor screening abnormalities	78 (43.3%)
Histology results in HPV HR12+ cases	180
Negative/LSIL/CIN1	142 (78.9%)
HSIL/CIN2+	38 (21.1%)
HSIL/CIN3+ *	12 (31.6%) *
Age (years)	
Min	25
Max	67
Median	33
Mean	34.7

Abbreviations: LBS, liquid-based screening; HRHPV, 14 high-risk types of human papillomavirus test; HPV 16/18, human papillomavirus types 16 and/or 18; HPV HR12, human papillomavirus 12 high-risk types other than types 16 and 18; DS, p16/Ki67 dual staining test; LBC, liquid-based cytology; NILM, negative for intraepithelial lesion or malignancy; ASC-US, atypical squamous cells of undetermined significance; LSIL, low-grade squamous intraepithelial lesion; ASC-H+, atypical squamous cells—cannot exclude HSIL or worse; LSIL/CIN1, histologic low-grade squamous intraepithelial lesion; HSIL/CIN2+, histologic high-grade squamous intraepithelial lesion with a quantification of cervical intraepithelial neoplasia in grade 2 or worse; HSIL/CIN3+, histologic high-grade squamous intraepithelial lesion with a quantification of cervical intraepithelial neoplasia in grade 3 or worse; min, minimum; max, maximum; *, % of HSIL/CIN2+; +, positive.

**Table 5:** HSIL/CIN2+ reporting rates and PPVs of different triage models for the detection of HSIL/CIN2+ in HPV HR12+ women.

	HSIL/CIN2+, No.	<HSIL/CIN2, No.	Total, No.	PPV, %
Triage of all HPV HR12+ with cytology alone (M1)				
NILM	(8)	(18)	26	-
ASC-US	12	50	62	19.4
LSIL	10	68	78	12.8
ASC-US/LSIL	22	118	140	15.7
ASC-H+	7	7	14	50.0
ASC-US/LSIL/ASC-H+	29	125	154	18.8
Triage of all HPV HR12+ with cytology and DS (M1A)				
NILM DS+	8	18	26	30.8
ASC-US DS+	12	17	29	41.4
LSIL DS+	10	24	34	29.4
NILM/ASC-US/LSIL DS+	30	59	89	33.7
ASC-H+	7	7	14	50.0
NILM/ASC-US/LSIL DS+/ASC-H+	37	66	103	35.9
NILM DS−	(0)	(0)	0	-
ASC-US DS−	(0)	(32)	32	-
LSIL DS−	(0)	(42)	42	-
NILM/ASC-US/LSIL DS−	(0)	(74)	74	-
Triage of all HPV HR12+ with DS alone (M2)				
DS+	34	68	102	33.3
DS−	(3)	(75)	78	-

Abbreviations: M1, Triage model 1; M1A, Triage model 1A; M2, Triage model 2; NILM, negative for intraepithelial lesion or malignancy; ASC-US, atypical squamous cells of undetermined significance; LSIL, low-grade squamous intraepithelial lesion; ASC-H+, atypical squamous cells—cannot exclude HSIL or worse; DS, p16/Ki67 dual staining test; HSIL/CIN2+, histologic high-grade squamous intraepithelial lesion with a quantification of cervical intraepithelial neoplasia in grade 2 or worse; HSIL/CIN2, histologic high-grade squamous intraepithelial lesion with a quantification of cervical intraepithelial neoplasia in grade 2; PPV, positive predictive value; +, positive; −, negative; (No) numbers in parentheses, potentially missed cases; there were no indications for colposcopy for these cases.

**Table 6:** Clinical performance with predictive values of different triage models for detection of HSIL/CIN2+ in HPV HR12+ women.

	HSIL/CIN2+, No.	<HSIL/CIN2, No.	Total, No.	PPV, % (95% CI)	NPV, % (95% CI)
Triage model with cytology alone (M1): ASC-US/LSIL/ASC-H+	29	125	154	18.8 (13.0, 25.9)	69.2 (48.2, 85.7)
Triage model with cytology and DS (M1A): NILM/ASC-US/LSIL DS+/ASC-H+	37	66	103	35.9 (26.7, 46.0)	100.0 (95.1, NA)
Triage model with DS alone (M2): DS+	34	68	102	33.3 (24.3, 43.4)	96.2 (89.2, 99.2)

Abbreviations: M1, Triage model 1; M1A, Triage model 1A; M2, Triage model 2; HPV HR12, human papillomavirus 12 high-risk types other than types 16 and 18; NILM, negative for intraepithelial lesion or malignancy; ASC-US, atypical squamous cells of undetermined significance; LSIL, low-grade squamous intraepithelial lesion; ASC-H+, atypical squamous cells—cannot exclude HSIL or worse; DS, p16/Ki67 dual staining test; HSIL/CIN2+, histologic high-grade squamous intraepithelial lesion with a quantification of cervical intraepithelial neoplasia in grade 2 or worse; HSIL/CIN2, histologic high-grade squamous intraepithelial lesion with a quantification of cervical intraepithelial neoplasia in grade 2; PPV, positive predictive value; NPV, negative predictive value; +, positive; CI, confidence interval.

**Table 7:** Comparison of predictive values of different triage models for detection of HSIL/CIN2+ in HPV HR12+ women.

	M1 (Cytology-Alone Triage)	M1A (Cytology and DS Triage)	*p*-Value
PPV for HSIL/CIN2+, % (95% CI)	18.8 (13.0, 25.9)	35.9 (26.7, 46.0)	<0.0001
NPV for HSIL/CIN2+, % (95% CI)	69.2 (48.2, 85.7)	100.0 (95.1, NA)	0.0024
	M1 (cytology-alone triage)	M2 (DS-alone triage)	
PPV for HSIL/CIN2+, % (95% CI)	18.8 (13.0, 25.9)	33.3 (24.3, 43.4)	<0.0001
NPV for HSIL/CIN2+, % (95% CI)	69.2 (48.2, 85.7)	96.2 (89.2, 99.2)	0.0079
	M1A (cytology and DS triage)	M2 (DS-alone triage)	
PPV for HSIL/CIN2+, % (95% CI)	35.9 (26.7, 46.0)	33.3 (24.3, 43.4)	0.0811
NPV for HSIL/CIN2+, % (95% CI)	100.0 (95.1, NA)	96.2 (89.2, 99.2)	0.0773

Abbreviations: M1, Triage model 1; M1A, Triage model 1A; M2, Triage model 2; HSIL/CIN2+, histologic high-grade squamous intraepithelial lesion with a quantification of cervical intraepithelial neoplasia in grade 2 or worse; PPV, positive predictive value; NPV, negative predictive value.

**Table 8:** Number of colposcopies needed to detect HSIL/CIN2+ and number of missed HSIL/CIN2+ cases in different triage models in primary HPV-based screening.

	No. of Colposcopies Needed in Each Model	No. of Colposcopies Needed to Detect One HSIL/CIN2+ Case	No. of Missed HSIL/CIN2+ Cases
Hypothetical model without triage (M0): HPV HR12+	180	4.74	0
Triage model with cytology (M1): ASC-US/LSIL/ASC-H+	154	5.31	8
Triage model with cytology and DS (M1A): NILM/ASC-US/LSIL DS+/ASC-H+	103	2.78	0
Triage model with DS only (M2): DS+	102	3.00	3

Abbreviations: M0, Hypothetical model 0; M1, Triage model 1; M1A, Triage model 1A; M2, Triage model 2; HPV HR12, human papillomavirus 12 high-risk types other than types 16 and 18; NILM, negative for intraepithelial lesion or malignancy; ASC-US, atypical squamous cells of undetermined significance; LSIL, low-grade squamous intraepithelial lesion; ASC-H+, atypical squamous cells—cannot exclude HSIL or worse; DS, p16/Ki67 dual staining test; HSIL/CIN2+, histologic high-grade squamous intraepithelial lesion with a quantification of cervical intraepithelial neoplasia in grade 2 or worse; +, positive.

## Data Availability

The data presented in this study are available on reasonable request from the corresponding author.

## Abbreviations

ASCCP 2019	2019 American Society for Colposcopy and Cervical Pathology risk-based guidelines
ASC-H	Atypical squamous cells, cannot exclude HSIL
ASC-H+	ASC-H or worse
ASC-US	Atypical squamous cells of undetermined significance
CB	Colposcopy with biopsy
CI	Confidence interval
DS	p16/Ki67 dual staining test
HPV 16/18+	HPV 16 and/or 18 positive results
HPV HR12	Human papillomavirus 12 high-risk types other than types 16 and 18
HPV HR12+	Human papillomavirus 12 high-risk types other than types 16 and 18 positive results
HRHPV	14 high-risk types of human papillomavirus tests
HSIL+	High-grade squamous intraepithelial lesions or worse
HSIL/CIN2+	Histologic high-grade squamous intraepithelial lesion with a quantification of cervical intraepithelial neoplasia in grade 2 or worse
HSIL/CIN3+	Histologic high-grade squamous intraepithelial lesion with a quantification of cervical intraepithelial neoplasia in grade 3 or worse
LBC	Liquid-based cytology
LBS	Liquid-based screening
LSIL	Low-grade squamous intraepithelial lesion
LSIL/CIN1	Histologic low-grade squamous intraepithelial lesion
max	Maximum
min	Minimum
M0	Hypothetical model 0
M1	Triage model 1
M1A	Triage model 1A
M2	Triage model 2
NILM	Negative for an intraepithelial lesion or malignancy
NPV	Negative predictive value
PPV	Positive predictive value
+	Positive
−	Negative
